# Environmental Parameters and Substrate Type Drive Microeukaryotic Community Structure During Short-Term Experimental Colonization in Subtropical Eutrophic Freshwaters

**DOI:** 10.3389/fmicb.2020.555795

**Published:** 2020-09-24

**Authors:** Changyu Zhu, David Bass, Yutao Wang, Zhuo Shen, Weibo Song, Zhenzhen Yi

**Affiliations:** ^1^Institute of Evolution and Marine Biodiversity, College of Fisheries, Ocean University of China, Qingdao, China; ^2^Pilot National Laboratory for Marine Science and Technology, Qingdao, China; ^3^Guangzhou Key Laboratory of Subtropical Biodiversity and Biomonitoring, School of Life Sciences, South China Normal University, Guangzhou, China; ^4^Department of Life Sciences, Natural History Museum, London, United Kingdom; ^5^Dongli Planting and Farming Industrial Co., Ltd., Lianzhou, China; ^6^Institute of Microbial Ecology and Matter Cycle, School of Marine Sciences, Sun Yat-sen University, Zhuhai, China; ^7^Southern Marine Science and Engineering Guangdong Laboratory (Zhuhai), Zhuhai, China

**Keywords:** biofilm, habitat, microeukaryotes, neutral process, species sorting, substrate

## Abstract

Microeukaryotes are key components of aquatic ecosystems and play crucial roles in aquatic food webs. However, influencing factors and potential assembly mechanisms for microeukaryotic community on biofilms are rarely studied. Here, those of microeukaryotic biofilms in subtropical eutrophic freshwaters were investigated for the first time based on 2,585 operational taxonomic units (OTUs) from 41 samples, across different environmental conditions and substrate types. Following conclusions were drawn: (1) Environmental parameters were more important than substrate types in structuring microeukaryotic community of biofilms in subtropical eutrophic freshwaters. (2) In the fluctuating river, there was a higher diversity of OTUs and less predictability of community composition than in the stable lake. Sessile species were more likely to be enriched on smooth surfaces of glass slides, while both free-swimming and attached organisms occurred within holes inside PFUs (polyurethane foam units). (3) Both species sorting and neutral process were mechanisms for assembly of microeukaryotic biofilms, but their importance varied depending on different habitats and substrates. (4) The effect of species sorting was slightly higher than the neutral process in river biofilms due to stronger environmental filtering. Species sorting was a stronger force structuring communities on glass slides than PFUs with more niche availability. Our study sheds light on assembly mechanisms for microeukaryotic community on different habitat and substrate types, showing that the resulting communities are determined by both sets of variables, in this case primarily habitat type. The balance of neutral process and species sorting differed between habitats, but the high alpha diversity of microeukaryotes in both led to similar sets of lifecycle traits being selected for in each case.

## Introduction

In the aquatic environment, surfaces of submerged materials often favor the attachment and eventual colonization by microorganisms, including bacteria, archaea, and microeukaryotes (Besemer, 2015). These organisms become enmeshed in a matrix of extracellular polymeric substances to form what is collectively known as “biofilm” (Sutherland, 2001). Biofilms are often referred to as microfouling, resulting in an undesirable accumulation of microorganisms (Zhang et al., 2014). They provide an effective strategy for microorganisms to survive in unfavorable environments and to colonize new niches (Hall-Stoodley et al., 2004). Hence, biofilms are considered to be good models for understanding processes governing the structure of communities in nature system. Mechanistic insight into community assembly is crucial to better understand the functioning of biofilms, which drive key ecosystem processes in water (Singer et al., 2010; Peter et al., 2011).

A small number of previous studies on biofilm community were published, mostly focusing on bacterial communities in various environments such as streams (Besemer et al., 2012), pools (Langenheder and Székely, 2011), lakes (Jackson et al., 2001), rivers (Lyautey et al., 2005), and the deep sea (Zhang et al., 2014). They indicated that environmental condition and substrate type were the most important factors in structuring bacterial communities (Lyautey et al., 2005; Besemer et al., 2012; Lee et al., 2014; Zhang et al., 2014). For instance, bacterial communities were mainly influenced by temperature, light, and hydrodynamic stability in Garonne River (Lyautey et al., 2005). Strong selection effect of the substrates on the microbial assembly was reported in the brine pool in Thuwal cold Seep (Zhang et al., 2014). By contrast, some studies showed that microbial community assembly can theoretically be dictated by neutral processes. In this model, random patterns in species co-occurrence and environmentally independent spatial autocorrelation (e.g., dispersal) were the main features of community structure if demographic stochasticity and limited dispersal alone were driving population dynamics, rather than species sorting (environmental filtering and interspecific competition) (Bell et al., 2005; Sloan et al., 2006; Bell, 2010; Langenheder and Székely, 2011; Li et al., 2019; Liu J. et al., 2019). For instance, the population dynamics of bacterial communities in the Palo Alto Regional Water Quality Control Plant were consistent with neutral community assembly (Ofiţeru et al., 2010). The neutral model also explained the distributions of bacterial communities of water-filled treeholes in large European beech trees (Woodcock et al., 2007). In addition, some investigations reported that both species sorting and neutral processes may shape the bacterial community structure, and their importance may differ depending on how many generalists and specialists are present in a community and homogenous condition of flow landscape (Langenheder and Székely, 2011; Woodcock et al., 2013; Wang et al., 2014; Li et al., 2018).

As predators, producers, decomposer, and parasites, microeukaryotes represent the bulk of microbial diversity and play key roles in the ecological functioning and process of aquatic biological ecosystems (Bik et al., 2012; Zhang et al., 2018; Zhao et al., 2018; Chen X. et al., 2019; Chi et al., 2019; Lu et al., 2019; Wang et al., 2020; Xu et al., 2020). Previous studies showed that several environmental factors play important roles in structuring microeukaryotic communities on biofilms. For instance, temperature, nutrients, and salinity were suggested as the strongest determinants of community structure of ciliates colonizing on glass slides in Jiaozhou Bay (Qingdao, China) (Gong et al., 2005). Apart from the effect of abiotic factors, predator–prey interactions between bacteria and eukaryotes were also identified as important factors in structuring morphology and function of biofilms from River Rhine in Cologne (Germany) (Wey et al., 2012). However, the extent to which different substrates determine the eukaryotic microbial communities growing on them remains unclear (Ragon et al., 2012; Cutler et al., 2013). For instance, green biofilm varied in association with major differences in limestone and sandstone in Belfast (Cutler et al., 2013). In contrast, algal community compositions were reported to have no significant correlation with substrate chemistry of exteriors of buildings in Europe and Latin America (Gaylarde and Gaylarde, 2005). Uher et al. (2005) found no connection between substrate type and algal communities on stone in southeastern Spain. Previous studies of microbial biofilms mostly concentrated on a single type of aquatic environment such as lakes (Xu et al., 2005; Jiang et al., 2007) or coastal seas (Xu et al., 2009; Abdullah Al et al., 2018; Sikder and Xu, 2020; Sikder et al., 2020). Few studies have compared microeukaryotic community assemblies on substrates in different aquatic fluctuating conditions (Battin et al., 2003; Besemer et al., 2007), even though it is recognized that current and tide play important role in microeukaryotic colonization (Xu et al., 2009). Additionally, mechanisms structuring microeukaryotic communities of biofilms on different substrates were rarely reported. We could find only one study showing that neutral process was the most influential process for microeukaryotic community assembly of epilithic biofilms on mineral composite substrates (Ragon et al., 2012). In summary, previous studies provided our understanding of microeukaryotic biofilms to some extent, but possible factors and potential mechanisms in structuring microeukaryotic community assembly on different aquatic fluctuating condition and substrates are still largely unknown.

Most biofilm-dwelling microeukaryotes are primary consumers and play an important role in controlling the transfer of energy to higher trophic levels in aquatic microbial food webs (Abdullah Al et al., 2018). Microeukaryotes are diverse and abundant in subtropical freshwater systems, because of high spatial and temporal heterogeneities (Chen W. et al., 2019). The Pearl River, the third biggest river in China, represents a diversity hotspot for microeukaryotes (Liu W. et al., 2019). The river is affected by tides from the Pearl River Estuary, presenting fluctuating biotic (seed planktonic microeukaryotic species) and abiotic environments (Huang et al., 2003) between river water at high and low tide. The Ming Lake, locating in Jinan University, is a lentic eutrophic lake, representing a stable aquatic environment. Hence, the Pearl River and Ming lake represent two typical subtropical freshwater environments, providing a good opportunity for investigating the microeukaryotic assembly on substrates under different conditions. We hypothesized that microeukaryotic communities of biofilms are significantly different in fluctuating river and stable lake environments. Polyurethane foam units (PFUs) and glass slides, which have been widely used in enrichment of microorganisms in aquatic ecosystems for biodiversity assessment (Cairns et al., 1969; Xu et al., 2005, 2009; Oberbeckmann et al., 2016). We predicted that microeukaryotic communities of biofilms may be assembled differently on these two substrates, considering that glass slides are smooth (two-dimensional) and PFUs possess many holes (three-dimensional).

In this study, microeukaryotic diversity of biofilms and water columns from the river and lake were investigated over time. We aimed to answer the following questions: (1) Do microeukaryotic communities of biofilms in different environment and substrates show similar diversity patterns? (2) Do environmental parameters or substrate types more strongly affect structuring microeukaryotic communities of biofilms?

## Materials and Methods

### Sample Collection

**FIGURE 1:**
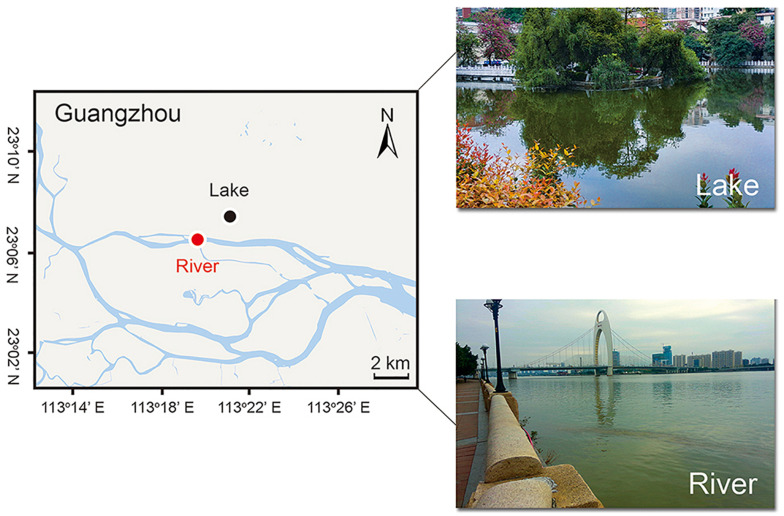
Location of sampling sites.

Microeukaryotic communities were collected in two sites: Ming Lake (23°13 N, 113°34 E, Guangzhou, China), a still freshwater lake, and Pearl River (23°11 N, 113°33 E, Guangzhou, China), the third biggest river in China (Figure 1). The sampling site in Pearl River is about 66 km away from river mouth, and semidiurnal tides are present. Temperature, pH, and dissolved oxygen (DO) were all measured for each sampling with an ORION 520M-01A (Thermo Fisher Scientific, MA, United States) multiparametric probe. About 0.1 L of water was collected at the depth of 30 cm below the surface water and then taken back to laboratory (South China Normal University, Guangzhou) within 1 h. In laboratory, total nitrogen (TN), total phosphorus (TP), ammonium nitrogen (AN), and chemical oxygen demand (COD) were measured using a DR3900 spectrophotometer (HACH, CO, United States) according to Water Analysis Handbook (HACH, CO, United States) (Supplementary Table S1).

Two types of artificial substrates were used to measure microeukaryotic communities: PFUs and glass slides. Glass slides offer a robust, inexpensive, and reliable method for allowing microeukaryotes to form biofilm and have been shown to harbor microeukaryotic species richness almost as high as those on natural substrates exposed to the same environmental conditions (Gong et al., 2005). The PFU method was standardized by the Environmental Protection Agency of China under number GB/T 12990-9 (Water Quality-Microbial Community Biomonitoring-PFU Method) [(SBTS) China State Bureau of Quality, and Technical Supervision, and (EPA) China State Environmental Protection Administration, 1992] for collecting microeukaryotes in aquatic ecosystems. Their effectiveness and practicability were validated by previous studies (Gong et al., 2005; Xu et al., 2005, 2009). In this study, the PFU method was based on standard protocol of Water Quality-Microbial Community Biomonitoring-PFU Method) [(SBTS) China State Bureau of Quality, and Technical Supervision, and (EPA) China State Environmental Protection Administration, 1992]. The PFU blocks were 6.5 × 6.5 × 7.5 cm in size and were soaked in distilled water for 24 h and squeezed before using. Ten glass slides were placed into a slide frame. Then the PFU blocks and glass slide frames were tied with thin ropes and placed at the depth of 30 cm below the surface water at the two sampling sides (Cairns et al., 1969). Biofilms on one piece of PFU and 10 pieces of glass slides were sampled by manual lifting. Water column samples (200 mL) were collected at the same depth as the biofilms. Previous studies (Plafkin et al., 1980; Xu et al., 2005, 2009) indicated that the microeukaryotic community would reach equilibrium within 28 days in lentic water and 15 days in flowing water. According to standard protocol of GB/T 12990-9 (Water Quality-Microbial Community Biomonitoring-PFU Method) [(SBTS) China State Bureau of Quality, and Technical Supervision, and (EPA) China State Environmental Protection Administration, 1992], sampling was done in Ming Lake at the 1st, 3rd, 7th, 11th, 15th, 21th, and 28th days (October 28–November 24, 2015) after the substrates were deployed, and in Pearl River on the 1st, 3rd, 7th, 11th, and 15th days (January 7–21, 2016). Water column samples were collected at both low tide and high tide. Totally, we collected 21 samples from Ming Lake and 20 samples from Pearl River (Supplementary Table S1).

Samples of the PFUs were obtained by manually squeezing as much water as possible, and those of glass slides were manually gently scraped in sterile water (approximately 200 mL). After that, three types of samples (PFUs, glass slides, and water columns) were filtered with a peristaltic pump (Vacuum Pump XF5423050; Millipore, MA, United States) through a 0.22-μm pore size polyethersulfone membranes (47-mm diameter; Pall, NY, United States). Then, the membranes were stored at −80°C until DNA extraction.

### DNA Extraction, Polymerase Chain Reaction, and High-Throughput Sequencing

Each membrane was cut by scissors and moved into bead tube. Then total DNA was extracted from the membranes using PowerSoil^®^ DNA Isolation Kit (MOBIO Laboratories, CA, United States) according to the manufacturer’s instructions. Total DNA was used as templates for polymerase chain reaction (PCR) amplification of the V4 region of the SSU rDNA (∼380 bp) using universal eukaryotic primers (Stoeck et al., 2010) TAReuk45FWD1 [5′-CCAGCA(G/C)C(C/T)GCGGTAATTCC-3′] and TAReuKREV3 [5′-ACTTTCGTTCTTGAT(C/T)(A/G)A-3′]. Each PCR reaction (20 μL) contained 5 × FastPfu buffer, 2.5 mM dNTPs, 1 U of FastPfu polymerase (TRANSGEN BIOTECH, Beijing, China), 5 μM of each primer, and 10 ng of target DNA. The amplification protocol consisted of an initial denaturation step of 95°C for 5 min, 27 cycles of denaturation at 95°C for 30 s, annealing at 55°C for 30 s, extension at 72°C for 45 s, and a final extension step at 72°C for 10 min. Then sequencing libraries were generated using TruSeq^®^ DNA PCR-Free Sample Preparation Kit for Illumina (San Diego, CA, United States) following manufacturer’s recommendations, and bar-code indexes were added. The library quality was assessed on the Qubit^®^ 2.0 Fluorometer (Thermo Fisher Scientific, MA, United States). Finally, PCR products were sequenced on an Illumina Hiseq instrument using a paired-end 250-bp sequence read run (Total Genomics Solution, Shenzhen, China).

### Sequence Analysis

The paired-end reads were merged with FLASH (Tanja and Salzberg, 2011). Raw sequence reads were analyzed and quality filtered in UPARSE v. 8.1 (Edgar, 2013), pipeline implemented in USEARCH v. 8.1 (Edgar, 2013), and QIIME v.1.8.0 (Caporaso et al., 2010). Sequences were filtered in order to generate high-quality reads through the QIIME quality-filtering pipeline (Caporaso et al., 2010). Sequences of length <200 or >500, average quality <20, ambiguous bases >0, or homopolymer length >6 were removed. Chimeras were identified and removed using UCHIME (Edgar et al., 2011). Remaining sequences were grouped into operational taxonomic units (OTUs) at a 97% similarity cutoff using the UPARSE default algorithms (Edgar, 2013). Afterward, singletons (OTUs with only one sequence) were discarded before the downstream analysis as potential sequencing errors. Then, we generated taxonomic assignment of the OTUs using SILVA 128 (Quast et al., 2012) using blast with default parameters within the QIIME program (Caporaso et al., 2010). Finally, to enable comparisons between samples, we used a randomly subsampled subset of 29,027 sequences from each sample to standardize sequencing effort across samples.

### Statistical Analysis

Statistical analysis and all graphic visualization were performed in R (R Core Team, 2019). In order to minimize outlier effects, logarithmic transformations were applied to the counts of reads attributed to each OTU and environmental factors (except for pH) for subsequent analyses. Richness and diversity of each sample were estimated by the total number of OTUs per sample and the “Shannon–Wiener” index, respectively, using the Vegan package (Oksanen et al., 2013). Venn diagrams were generated to show shared numbers of OTUs between the different sample types (glass slide, PFU, and water column) within the same environment and same sample type from different environment using the “VennDiagram” package (Chen and Boutros, 2011). Bray–Curtis dissimilarity matrix, which is considered to be one of the most robust dissimilarity coefficients for ecological studies (Kent, 2011), was applied to our microeukaryotic OTU relative abundance of all samples. In order to compare the relative species abundance among different samples, a heatmap was generated using the “gplots” package (Warnes et al., 2009).

Non-metric multidimensional scaling (NMDS) analysis was performed on the Bray–Curtis dissimilarity matrix to visualize patterns of community composition, and the significant differences between sample types were tested by running a permutational multivariate analysis of variance (ADONIS) (Anderson, 2001; Clarke et al., 2008; Yoshioka, 2008). Redundancy analysis (RDA) was performed to explore the relationships between microeukaryotic communities of sample types and water environmental factors. This method was chosen because preliminary detrended correspondence analysis on microeukaryotic communities revealed that the longest gradient lengths were shorter than 3.0, indicating that the majority of species exhibited a linear response to the environmental variation (Lepš and Šmilauer, 2003). The significance of the axes obtained by the RDA was determined based on the Monte Carlo permutation test (Manly, 2006). A Kruskal–Wallis test (Breslow, 1970) was performed to test significant difference for α-diversities and environmental factors between lake and river, water at high tide and low tide, and α-diversities among different sample types within the same environment. A neutral community model (NCM) was used to determine the potential importance of neutral processes on community assembly of different biofilms in different environment (Sloan et al., 2006). The NCM is an adaptation of Hubbell’s NCM (Hubbell, 2001) adjusted to large microbial populations analyzed with molecular tools (Sloan et al., 2006). NCM is used to determine the potential contribution of neutral processes (such as dispersal and ecological drift) to community assembly by predicting the relationship between OTU occurrence frequency and their relative abundance (Sloan et al., 2006). In this model, *R*^2^ represents the overall fit to the neutral model; the Nm indicates the metacommunity size (N) times immigration (m). The confidence is 95%, based on 1,000 bootstrap replicates.

## Results

### Comparison of Diversity and Community Composition

**FIGURE 2:**
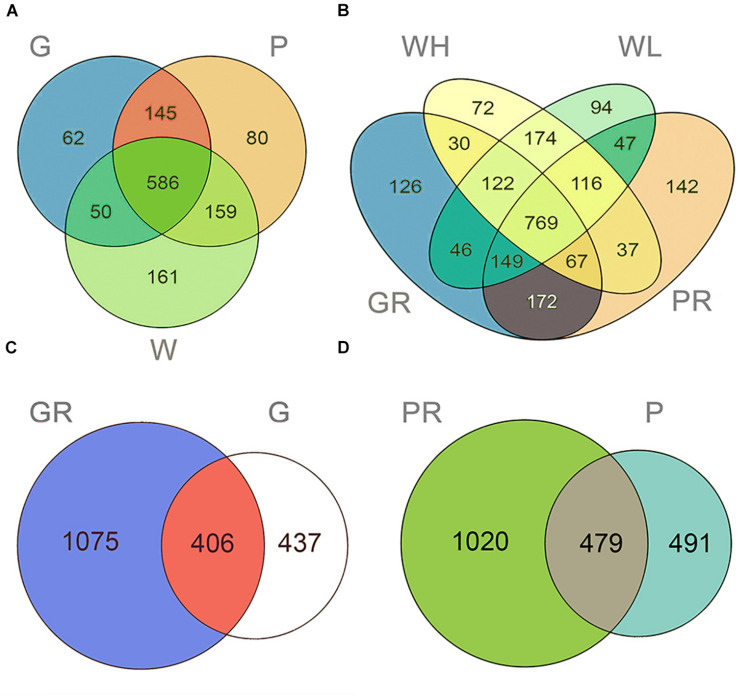
Venn diagrams displaying the number of unique and shared OTUs in different samples of Ming Lake **(A)** and Pearl River **(B)** and the number of unique and shared OTUs in different environment of glass slides **(C)** and PFUs **(D)**. Samples are named as follows: G, glass slides in Ming Lake; P, PFUs in Ming Lake; W, water columns in Ming Lake; GR, glass slides in Pearl River; PR, PFUs in Pearl River; WH, water columns at high tide in Pearl River; WL, water columns at low tide in Pearl River. Detailed information of samples is given in Supplementary Table S1.

**FIGURE 3:**
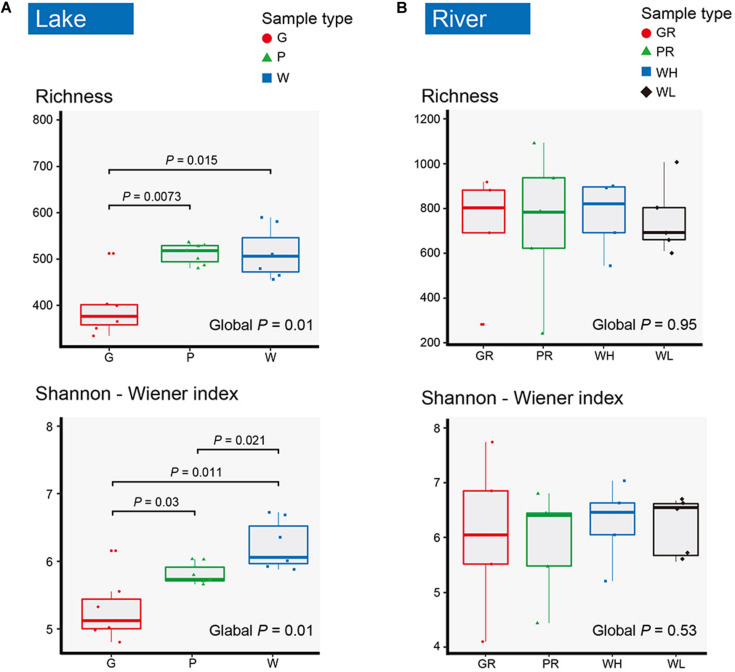
The boxplots for richness and Shannon–Wiener index of microeukaryotes in lake **(A)** and river **(B)**. Samples are named as follows: G, glass slides in Ming Lake; P, PFUs in Ming Lake; W, water columns in Ming Lake; GR, glass slides in Pearl River; PR, PFUs in Pearl River; WH, water columns at high tide in Pearl River; WL, water columns at low tide in Pearl River. The global *P*-value represents the significance among sample types in lake and river. The significant *P*-value between two sample types is shown (*P* < 0.05). Significance tests were based on Kruskal–Wallis test.

In total, 2,585 OTUs were detected across all samples. There were 1,259 and 2,299 OTUs detected in lake and river samples, respectively (Figures 2A,B). Among them, 620 (49.25%) and 813 (35.36%) were shared among three sample types (water columns, glass slides, and PFUs) in lake and river samples, respectively (Figures 2A,B). For the substrate sample types, totals of 1,918 OTUs and 1,990 OTUs were detected in glass slide (Figure 2C) and PFU samples (Figure 2D), respectively. Of these, 406 OTUs (21.17%) were shared between glass slide samples from the lake and river, and 479 (24.07%) were shared between PFU samples from the lake and river. Both richness and Shannon–Wiener indices among three sample types were significantly different overall (*P* < 0.05) in the lake samples, but not in the river samples (Figure 3). Comparing samples from the lake and river (Supplementary Figure S1A), both richness and Shannon–Wiener indexes of all samples in the river were significantly higher than those of the lake. However, there were no significant α-diversity differences between all water column samples in river at high tide and low tide (Supplementary Figure S1B).

**FIGURE 4:**
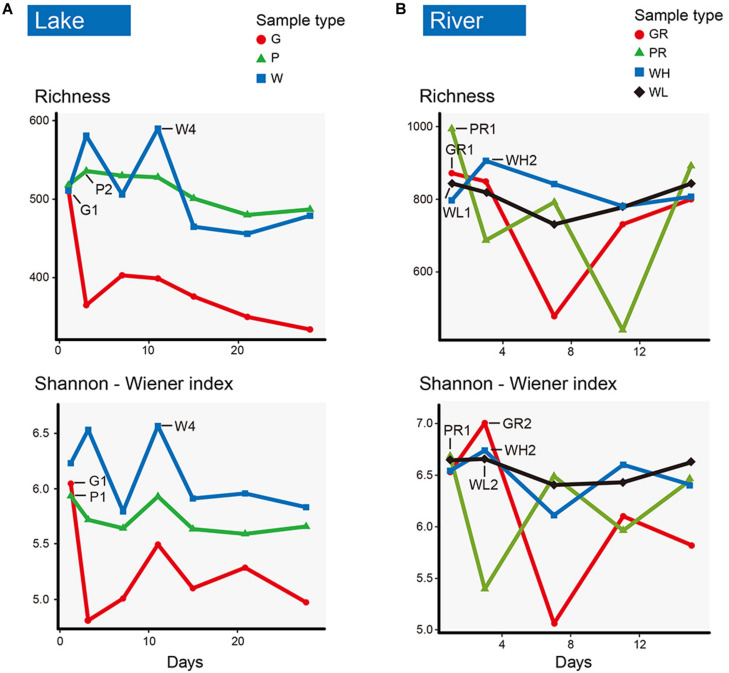
Richness and Shannon–Wiener index of microeukaryotic communities from glass slides (G, red), PFUs (P, green), and water columns (W, blue) in lake **(A)** across sampling time (1st, 3rd, 7th, 11th, 15th, 21th, 28th days). Richness and Shannon–Wiener index of glass slides (GR, red), PFUs (PR, green), water columns (WH, blue) at high tide, and water columns at low tide (WL, black) in river **(B)** across sampling time (1st, 3rd, 7th, 11th, 15th days).

**FIGURE 5:**
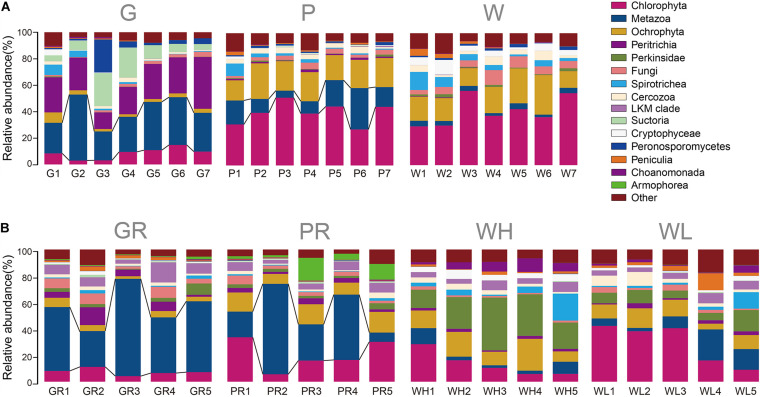
Taxonomic profile of microeukaryotic OTUs of every sample type from the lake **(A)** and river **(B)** over time. Samples are named as follows: G, glass slides in Ming Lake; P, PFUs in Ming Lake; W, water columns in Ming Lake; GR, glass slides in Pearl River; PR, PFUs in Pearl River; WH, water columns at high tide in Pearl River; WL, water columns at low tide in Pearl River. The relative abundance of each group >1% is shown.

Of the 21 lake samples, the highest richness and the highest Shannon–Wiener index occurred in the water sample on the 11th day (W4). The richness of glass slide and PFU richness levels were highest on day 1 (G1) and day 3 (P2), respectively. The Shannon–Wiener diversity levels of both glass slides and PFUs were highest on day 1 (G1, P1), but always lower than water. The richness and diversity of PFUs were consistently higher than those on glass slides (Figure 4A). Of the 20 river samples, richness and Shannon–Wiener diversity indices of PFUs and glass slides showed much greater variation than in the lake, sometimes exceeding that of the water samples (Figure 4B). The richness of samples from glass slides and PFUs were highest on the 1st day (GR1, PR1). The highest Shannon–Wiener index of samples from glass slides and PFUs occurred on the 3rd day (GR2) and the 1st day (PR1), respectively. The variability of microeukaryotic assemblages was greater across the sampling period in the river samples than in the lake (Figure 5). For example, the changes in relative abundance of Metazoa on GR (SD = 0.17) and in water (0.25) were greater than that of lake (SD = 0.10 and 0.09, respectively).

**FIGURE 6:**
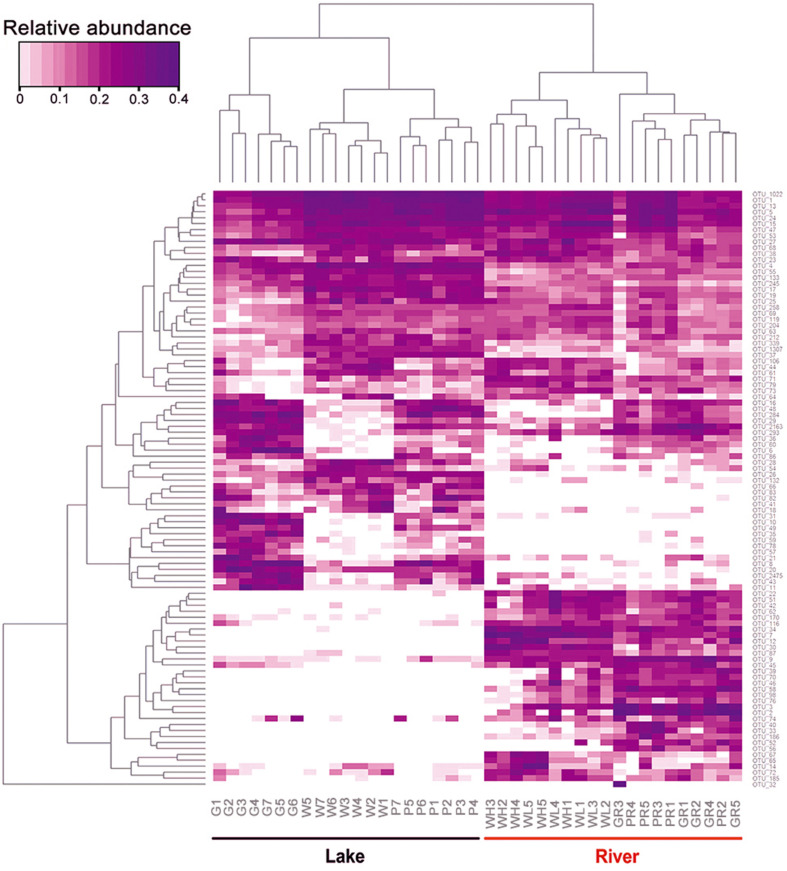
Relative abundance of top 100 microeukaryotic OTUs with most abundant read counts. Samples are labeled in lateral axis, and abundance of each OTU is labeled in vertical axis. The phylogenetic tree was calculated using neighbor-joining method, and the relationship among samples was determined by Bray distance. The relative abundance for each OTU was depicted by color intensity with the legend indicated at the left top of the figure. Samples are named as follows: G, glass slides in Ming Lake; P, PFUs in Ming Lake; W, water columns in Ming Lake; GR, glass slides in Pearl River; PR, PFUs in Pearl River; WH, water columns at high tide in Pearl River; WL, water columns at low tide in Pearl River.

**FIGURE 7:**
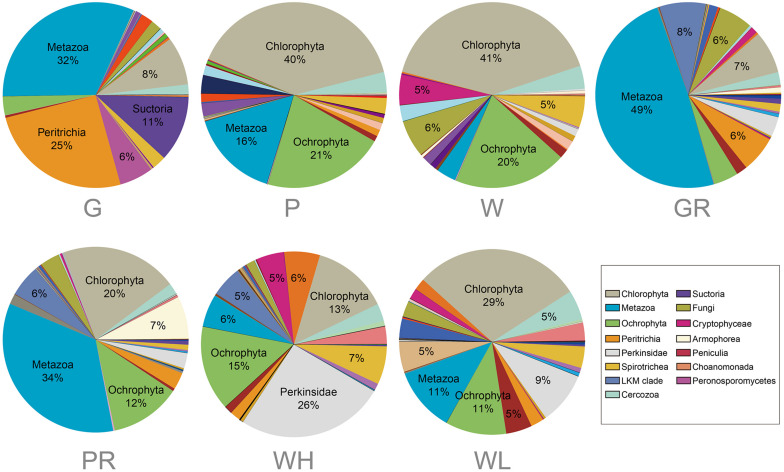
Comparison of taxonomic profile of microeukaryotic OTUs among different sample types from the lake and river. Samples are named as follows: G, glass slides in Ming Lake; P, PFUs in Ming Lake; W, water columns in Ming Lake; GR, glass slides in Pearl River; PR, PFUs in Pearl River; WH, water columns at high tide in Pearl River; WL, water columns at low tide in Pearl River. The relative abundance of each group >5% is shown. Abundant groups represented by >10% of each taxonomic unit were labeled with group name, and the relative abundance of each group >5% was labeled with percentage in pie charts.

A heatmap showing the occurrence of the top 100 most frequently detected OTUs showed a strong distinction between river and lake samples (Figure 6). The 41 samples were divided into two clades: clade “Lake” containing all 21 samples from Ming Lake and clade “River” containing all 20 samples from Pearl River. Within the lake clade, microeukaryotic communities clustered according to sample types: the water and PFU clusters being more closely related to each other than either to the glass slides. Chlorophyta (Archaeplastida) and Ochrophyta (Stramenopiles) were relatively abundant in water (41 and 20%, respectively) and PFU samples (40 and 21%, respectively) but rare on glass slides (<10%) (Figure 7). In contrast, OTUs annotated as peritrich and suctorian ciliates (Alveolata), with a primarily sessile lifestyle, were abundant in biofilm samples of glass slides (25 and 11%, respectively) but rare in water and PFUs (lower than 1%). The river clade comprised two subclades, one containing two clusters of water samples, one mostly (4/5) high tide, the other mostly (4/5) low tide. The other subclade also comprised two clusters, one mostly (4/5) from PFU samples, and the other mostly (4/5) from glass slides. OTUs assigned to Metazoa (Opisthokonta) were abundant in biofilm samples of PFUs (34%) and glass slides (49%), whereas they were moderate in water columns (10% for high tide and 14% for low tide, respectively). In contrast, Perkinsidae (Alveolata) was abundant in samples of water columns compared to those from glass slides and PFUs.

### Dissimilarity Among Communities

**FIGURE 8:**
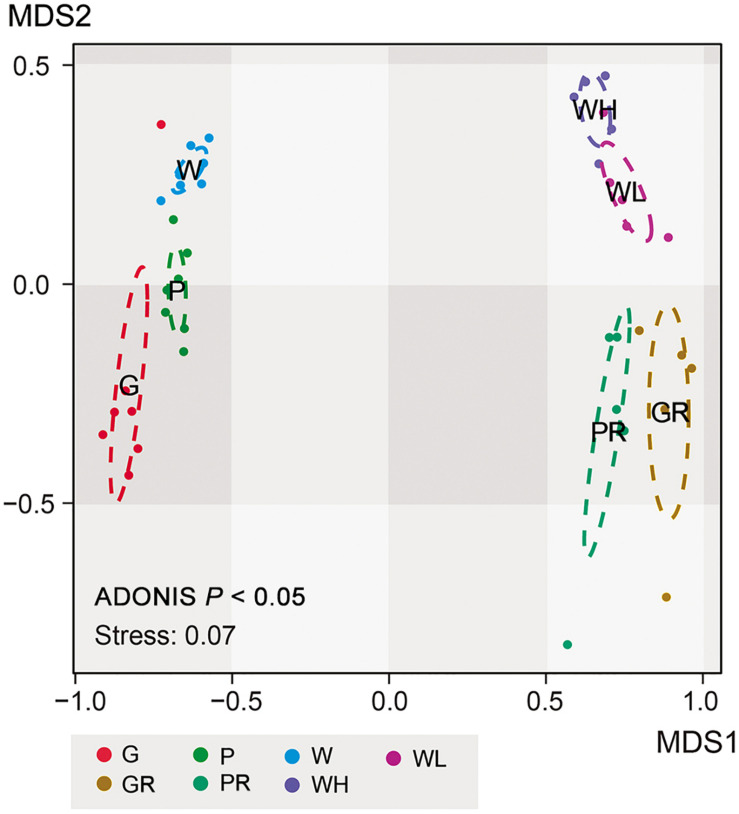
NMDS ordination of 21 samples in Ming Lake and 20 samples in Pearl River, showing clustering into groups according to environments (Ming Lake or Pearl River) and sample types (glass slides, PFUs and water columns). G, glass slides samples from lake; P, PFU samples from lake; W, water column samples from lake; GR, glass slides samples from river; PR, PFU samples from river; WH, water column samples at high tide from river; WL, water column samples at low tide from river. The ellipses represent 95% confidence intervals. *P* represents significance between every two sample types based on ADONIS analysis.

Non-metric multidimensional scaling ordinations showed strong separation of lake and river communities (Figure 8), consistent with the heatmap (Figure 6); all sample types clustered significantly separately from each other (ADONIS; *P* < 0.05; Figure 8). And the distance/community dissimilarity between biofilms and water columns were shorter/lower in lake than in river, indicating that the habitat type played an important role in dissimilarities between biofilms and water columns. Additionally, in the river, the state of the tide also played an important role in modifying the microeukaryotic assemblages.

### The Effect of Environmental Factors on Microeukaryotic Community Structures

Temperature, pH, and DO in the lake were significantly higher than those in river, whereas the TP, TN, and AN in river were significantly higher than those in lake (*P* < 0.05, Table 1). The mean value of AN in river (10.01) was approximately 33 times of that in lake (0.30). Further, environmental factors of river water at high tide were significantly different from low tide except for temperature. COD was the most different factor between river at high tide and river at low tide (three times).

**TABLE 1:** The comparisons for difference of environmental factors among lake, river, river at high tide, and river at low tide.

Comparison	Lake vs. river		Lake vs. river at low tide		Lake vs. river at high tide		High tide vs. low tide	
				
Factor/mean value	Lake	River	Lake	River at low tide	Lake	River at high tide	River at high tide	River at low tide
Temperature	25.66*	18.77*	25.66*	18.7*	25.66*	18.84*	18.84	18.7
DO	5.23*	0.98*	5.23*	0.45*	5.23*	1.50*	1.50*	0.45*
pH	7.27*	6.91*	7.27*	6.96*	7.27*	6.86*	6.86	6.96
COD	31.39	52.73	31.39*	81.98*	31.39	23.48	23.48*	81.98*
TP	0.28*	0.95*	0.28*	1.23*	0.28*	0.68*	0.68*	1.23*
TN	1.53*	12.15*	1.53*	15.30*	1.53*	9.00*	9.00*	15.30*
AN	0.30*	10.01*	0.30*	14.02*	0.30*	6.00*	6.00*	14.02*

Numbers represent mean value of environmental factors. *P < 0.05 is considered as significant effect.

Redundancy analysis revealed significant relationships between variation in environmental factors and microeukaryotic communities (Supplementary Figure S2). The variance between microeukaryotic communities explained by the first two axes of RDA was 56.7% for water column samples, 60.6% for biofilm samples of glass slides, and 63.8% for biofilm samples of PFUs. A Monte Carlo permutation test (Table 2) also a revealed a significant (*P* < 0.05) relationship between the environmental factors and community structures. For the glass slide samples, the correlation coefficient between AN and microeukaryotic community structures was highest of all sample types (0.97). For PFU samples, the highest correlation coefficient was 0.97, which was contributed by TN. For water column samples, AP showed the strongest correlation (0.92) with microeukaryotic community structures. Although the environmental factors with the highest correlation coefficient were different among three sample types, nutrients (TP, TN, and AN) have a strong correlation with microeukaryotic community structures of glass slides (0.95 on average), PFUs (0.96 on average), and water columns (0.85 on average).

**TABLE 2:** The correlation coefficient between the microeukaryotic community structure and environmental factors and result of Monte Carlo permutation test in different sample types.

Sample	Glass slides		PFUs		Water columns	
			
types/						
factors	*r*^2^	*P*	*r*^2^	*P*	*r*^2^	*P*
Temperature	0.77**	0.002	0.89***	0.001	0.82***	0.001
DO	0.77**	0.002	0.81**	0.002	0.83***	0.001
pH	0.58*	0.018	0.65**	0.007	0.73**	0.002
COD	0.76**	0.002	0.74**	0.008	0.88***	0.001
TP	0.92**	0.002	0.96**	0.002	0.92***	0.001
TN	0.97**	0.002	0.97**	0.002	0.80***	0.001
AN	0.97***	0.001	0.96**	0.002	0.82***	0.001

Significant codes: ***P ≤ 0.001; **P ≤ 0.01; *P ≤ 0.05. Permutation: free. Number of permutations: 1,000.

### The NCM Partly Explains Community Variation

**FIGURE 9:**
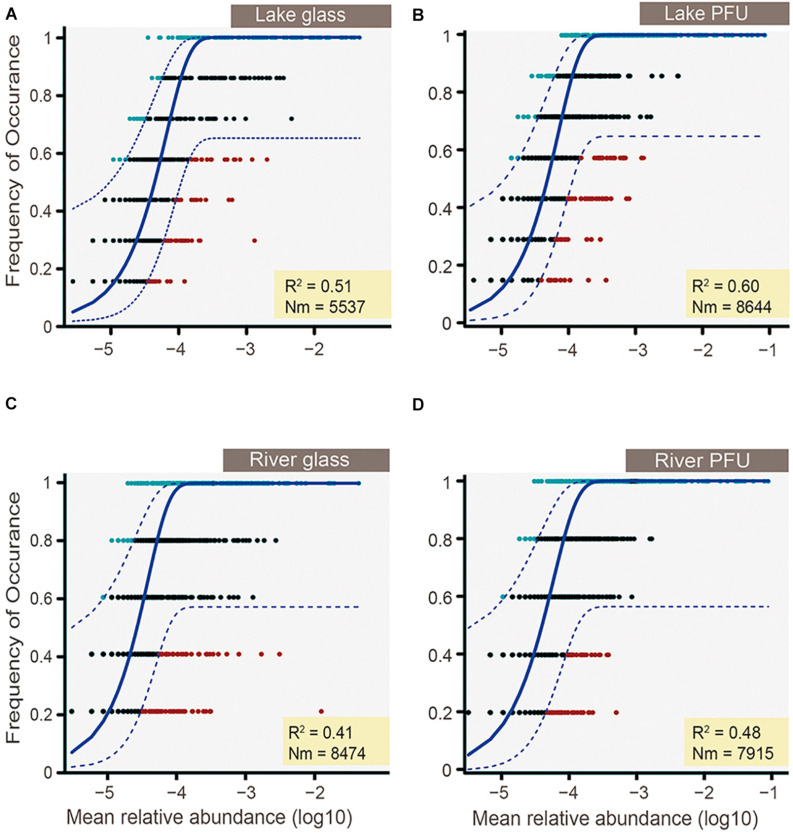
Frequency of occurrence of OTUs as a function of mean relative abundance (logarithmic transformed) for lake glass **(A)**, lake PFUs **(B)**, river glass **(C)**, and river PFUs **(D)**. The solid blue line indicates the best fit to the neutral model as in Sloan et al. (2006), and the dashed blue line represents 95% confidence intervals around the model prediction. Different colors represent OTUs with more or less frequency than predicted. Nm indicates metacommunity size times immigration. *R*^2^ indicates the fit to the neutral model, and negative *R*^2^ values indicate no fit to this model.

The NCM was used to determine the potential importance of neutral processes on community assembly of different biofilms in different environment. The NCM explained nearly 50% of taxon detection frequency in both lake and river (Figure 9), indicating that both species sorting and neutral process played an important role in microeukaryotic biofilm assembly. Furthermore, the NCM explained >50% of taxon detection frequency from the lake (51% for glass slides and 60% for PFUs) (Figures 9A,B), but <50% in the river (41% for glass slides and 48% for PFUs) (Figures 9C,D). Thus, the neutral process exerted slightly more effect on the lake than the river. The NCM also explained more variation in PFU samples (60% for lake and 48% for river) (Figures 9B,D) than glass slides counterparts (51% for lake and 41% for river) (Figures 9A,C).

## Discussion

### Comparing Microeukaryotic Diversity Between Habitats and Sample Types

The microeukaryotic diversity (water and biofilms) of river samples was higher overall than that of lake samples (Figure 3 and Supplementary Figure S1). High abundance of microeukaryotes in river samples can be attributed to a fluctuating environment, providing a wider range of ecological conditions and greater opportunities for microorganisms to successfully establish on substrates than the lake, as also indicated by a previous study (Besemer et al., 2007). In addition, flow velocity can also promote the flux of microorganisms from the bulk liquid to biofilms (Besemer et al., 2007). The Pearl River is composed of many tributaries from different ecological conditions (Huang et al., 2003), providing a diverse set of species from many niches to potentially colonize substrates, resulting in higher microeukaryotic diversity both in water columns and biofilms from the river than in the lake. Another reason for elevated diversity may be the high load of anthropogenic nutrients into Pearl River from increased agricultural activities, fish dike farming, and wastewater runoff due to the increase in population and economic development along the river (Huang et al., 2003), considering that nutrients (TP, TN, and AN) have strong correlation with microeukaryotic community structures (Table 2). It has been demonstrated that nutrients could directly affect photosynthesis by autotrophic microeukaryotes in water columns, and then phytoplankton growth impacts abundance of heterotrophic ones (Wang et al., 2014). A previous investigation also revealed that bacterial diversity in tropical stream biofilms increased with nitrate concentrations (Burgos-Caraballo et al., 2014).

Operational taxonomic units affiliated to Metazoa and Perkinsidae were more abundant in river samples than in lake (Figure 7), raising the possibility of a parasitic relationship between them in the river. Perkinsidae is group of parasitic microeukaryotes (Adl et al., 2019), which is hosted by metazoans such as bivalves, frogs, and fish (Chambouvet et al., 2015; Freeman et al., 2017).

In the present study, a large algal diversity in different sample types was detected. This is consistent with previous studies showing that algae were the most important primary producers in freshwater (Besemer et al., 2007). Among the three sample types, biofilms on glass slides contained a high proportion of sessile ciliates (Peritrichia) (Figure 7). This is concordant with previous studies showing that glass slides as artificial substrates allow microorganisms to form a periphyton or biofilm, in which periphytic ciliates (especially peritrich ciliates) were usually in high abundance and richness (Cairns and Yongue, 1968). The glass slides also hosted a high proportion of metazoans (Figure 7), in agreement with a previous study showing that Metazoa was the most abundant phylum on glass slides from Jauron River (Bricheux et al., 2013). However, our PFUs hosted a high portion of algae (Chlorophyta and Ochrophyta), which was inconsistent with previous studies showing that flagellates and ciliates were abundant on this substrate (Xu et al., 2005; Jiang et al., 2007). One explanation is that the high nutrient concentration in our sampling region leads to the high proportion of algae, which was also demonstrated in previous investigations (Biggs, 2000).

### Factors and Potential Mechanisms Influencing Microeukaryotic Community Structures

Both the heatmap (Figure 6) and NMDS (Figure 8) revealed that environmental parameters exerted stronger effects on microeukaryotic communities than sample type in our study. The Ming Lake is a small closed lake (Figure 1 and Supplementary Table S1), representing a stable aquatic environment, while the Pearl River represents a typical fluctuating river environment about 66 km far away from the river mouth (Figure 1 and Supplementary Table S1), subject to semidiurnal tides. Variation in microeukaryotic α-diversities and community compositions were greater in the river than the lake (Figures 4, 5). There could be several explanations for this. In the river, environmental factors and water flow direction differed daily at low and high tide (Table 2), resulting in diverse environmental stresses on the microbial communities (Böer et al., 2009; Zhang et al., 2020). Changing tides can affect biotic factors such as source communities and species interaction. This may result from “seed” planktonic microeukaryotic species from upstream and downstream being different and possible increased competition between microbes due to tidal fluctuation (Lee et al., 2011, 2014). Tidal activity is also likely to influence the structure and function of bacterial community in tidal habitats (Lv et al., 2016). Both abiotic (environmental factors) and biotic factors (source community and species interaction) play important roles in shaping microeukaryotic communities.

Within a given habitat, substrate types shaped microeukaryotic community assembles. The heatmap (Figure 6) and NMDS analyses (Figure 8) showed that within both lake and river environments, community compositions of biofilms generally grouped according to type of substrates (PFUs, slides), as well as sampling time. Some previous investigations studying bacterial and archaeal communities also recognized substrate type as a key factor affecting microbial community compositions (Sundberg et al., 2013; Ziganshin et al., 2013; Kalenitchenko et al., 2015). Obviously, properties of the substrates influenced microeukaryotic adhesion as has been shown for bacterial adhesion (Bernardes et al., 2012). In our study, those species with strong adhesion (sessile type) were more likely to be enriched on smooth surfaces of glass slides (Figure 7), on which other species are less able to live (Gong et al., 2005). By contrast, both free-swimming and attached organisms can easily colonize PFUs (Figures 5, 7) due to the holes and increased surface area inside the PFU, through which water can flow, but more slowly than across glass slides. In general, the smooth surfaces of glass slides more strongly selected specific groups of microeukaryotes than PFUs (Gong et al., 2005).

The community structures of biofilms from the lake and river were driven by both species sorting and neutral processes (Figure 9). Previous studies also showed that the relative importance of both species sorting and neutral processes changes not only across scales, but also during biofilm succession (Gong et al., 2005; Ragon et al., 2012). In our study, species sorting can be characterized by the effects of environmental parameters (environmental factors and tide) and substrates. Neutral process can be characterized by immigration and emigration of microeukaryotes on substrates. Selective pressures of environmental parameters and substrate have effects on microeukaryotic biofilms, but no overwhelming effect was detected, in agreement with a previous study that showed substrate characteristics impact the abundance/biomass but not microeukaryotic diversity (Cutler et al., 2013).

Interestingly, the effect of species sorting was slightly higher than the neutral process in river biofilms, whereas the opposite applied to the lake samples (Figure 9). We suggest this is due to stronger environmental filtering in the river, with its constantly changing conditions (tide and environmental factors). On the other hand, water flow in the river likely increases dispersal, increasing neutral processes relative to species sorting. In the relatively stable lake, environmental fluctuations were lower, and therefore neutral processes more strongly influenced community assembly. Although dispersal effects in the lake were likely lower than in the river, local alpha diversity of microeukaryotes is known to be very high relative to spatially more expansive levels of diversity (beta, gamma), and therefore the local “pool” of available lineages to colonize the different substrates is very large, so that the differences between substrate type are likely to be the strongest determinant of community assembly in the lake. Concordantly, the overall effect of species sorting was slightly lower for PFUs than glass slides, as PFUs provide a greater diversity of niches for microeukaryotes. In contrast to glass slides, which are generally only suitable for sessile taxa (Gong et al., 2005), PFUs can host planktonic, periphytic, and benthic protozoan assemblages and have relative large surface areas for colonization of sessile eukaryotes and lacunae, which can host planktonic forms (Lugo et al., 1998).

## Conclusion

As the first investigation focusing on influencing factors and potential assembly mechanisms for microeukaryotic community on biofilms in subtropical eutrophic freshwaters, we revealed the relative importance of species sorting and neutral processes for microeukaryotic community assembly on different substrates, and how this differed between a stable (lake) and fluctuating (tidal river) environment. The effect of species sorting was slightly higher than neutral processes in river biofilms due to stronger environmental filtering. Sessile species were more likely to be enriched on smooth surfaces of glass slides, while both free-swimming and attached organisms occurred within holes inside PFUs. Species sorting was enhanced on glass slides relative to PFUs, which have a greater diversity of niches. Environmental parameters such as environmental factors and tidal effects were more important than substrate types in structuring microeukaryotic community of biofilms in both lake and river. To test these preliminary results further, a more highly replicated study is now justified, both at individual time points and across a longer period of time.

## Data Availability Statement

All Illumina sequencing datasets are deposited in the NCBI under the accession number PRJNA623677.

## Author Contributions

ZY conceived the study. CZ carried out the experiments and performed the data analyses. CZ, ZY, and DB primarily wrote this manuscript. YW, ZS, and WS prepared the figures and participated in discussion. All authors agreed to be held accountable for the work performed therein.

## Conflict of Interest

YW was employed by Dongli Planting and Farming Industrial Co., Ltd. The remaining authors declare that the research was conducted in the absence of any commercial or financial relationships that could be construed as a potential conflict of interest.

## Supplementary Material

FIGURE S1 The boxplots for richness and Shannon-Wiener index of between different environment (Lake vs. River) and different water columns in river (WH vs. WL).

FIGURE S2 RDA ordination showing the microbial eukaryotic community structures of glass slides **(A)**, PFUs **(B)** and water columns **(C)** in lake and river in relation to environmental factors. Tem, temperature; pH; DO, dissolved oxygen; TN, total nitrogen; TP, total phosphorus; AN, ammonia nitrogen; COD, chemical oxygen demand. The black characters represent samples, blue arrows represent measured environmental factors.

TABLE S1 The information of samples (sample ID, Sample Type, Date Location, Latitude and longitude and environmental Factors).
