# Supplementary figures and images for: Environmental Parameters and Substrate Type Drive Microeukaryotic Community Structure During Short-Term Experimental Colonization in Subtropical Eutrophic Freshwaters
Source: Front Microbiol. 2020 Sep 24;11:555795. doi: 10.3389/fmicb.2020.555795 (PMC7541896; doi:10.3389/fmicb.2020.555795)

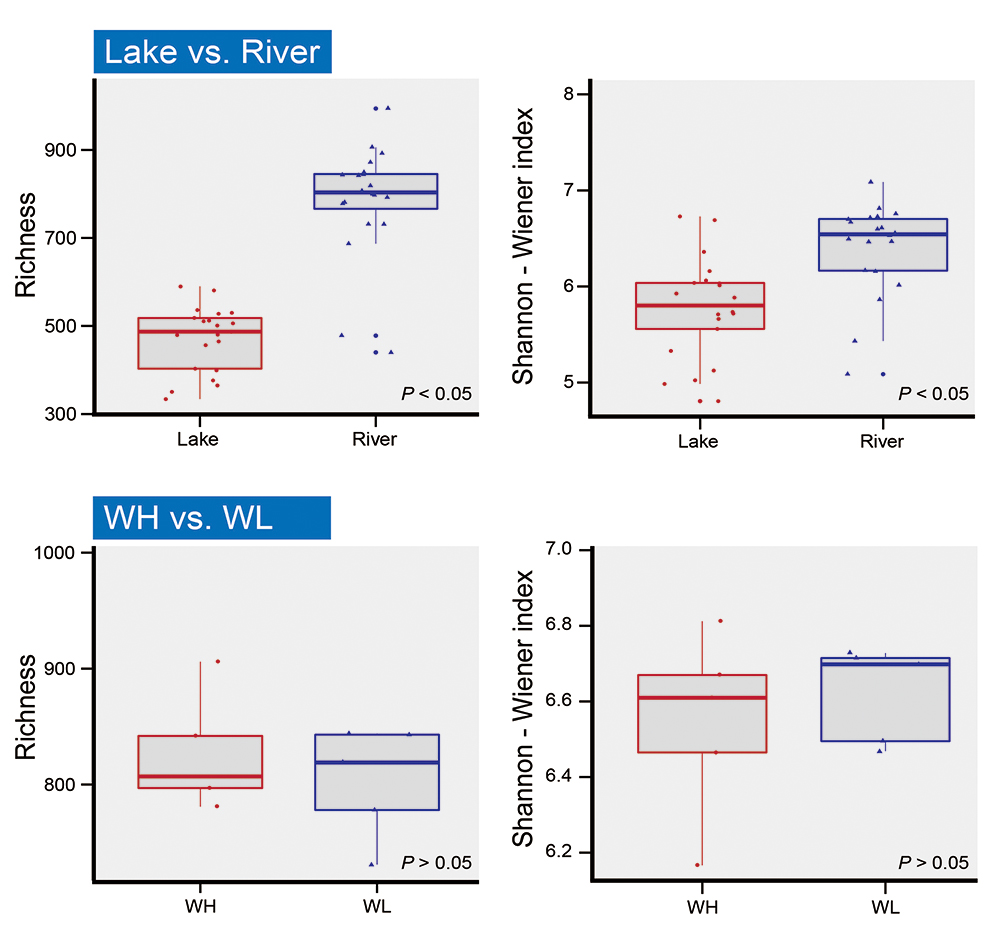

Supplement: FIGURE S1 — The boxplots for richness and Shannon-Wiener index of between different environment (Lake vs. River) and different water columns in river (WH vs. WL). [file Image_1.JPEG]

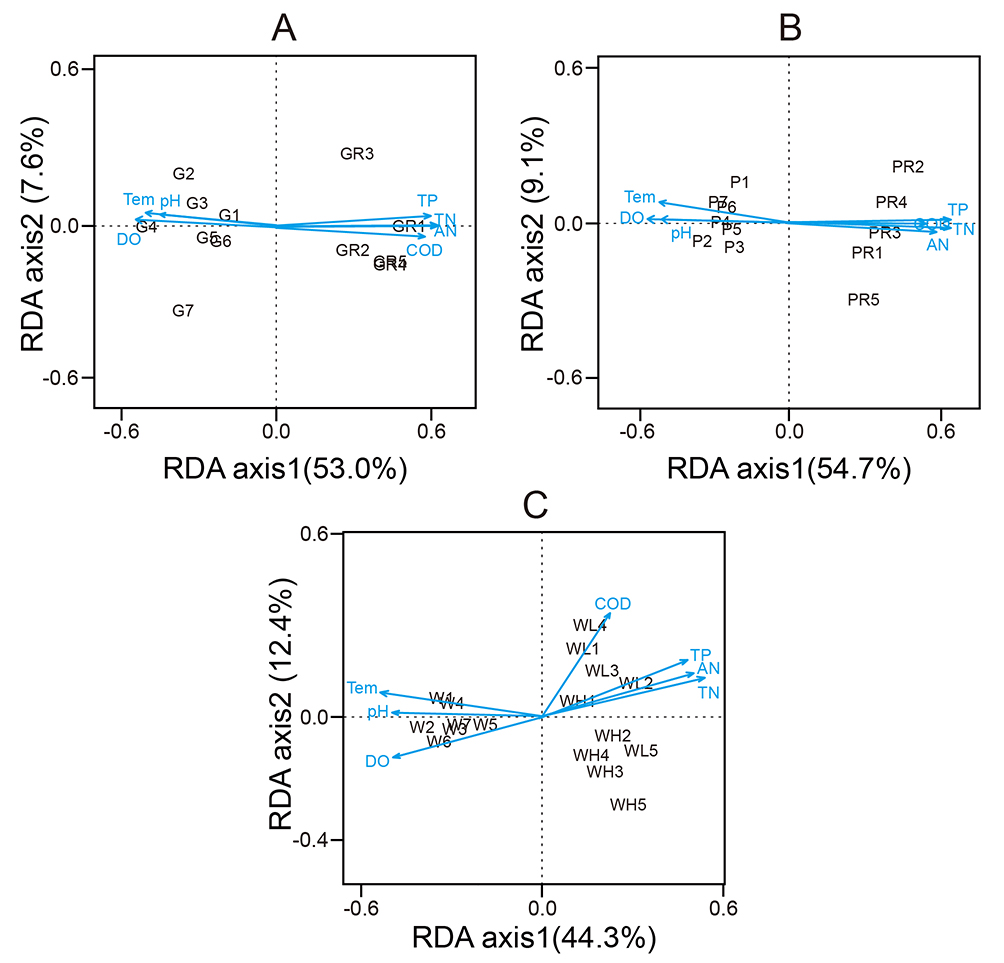

Supplement: FIGURE S2 — RDA ordination showing the microbial eukaryotic community structures of glass slides (A), PFUs (B) and water columns (C) in lake and river in relation to environmental factors. Tem, temperature; pH; DO, dissolved oxygen; TN, total nitrogen; TP, total phosphorus; AN, ammonia nitrogen; COD, chemical oxygen demand. The black characters represent samples, blue arrows represent measured environmental factors. [file Image_2.JPEG]
