# Supplementary material for: Environmental Parameters and Substrate Type Drive Microeukaryotic Community Structure During Short-Term Experimental Colonization in Subtropical Eutrophic Freshwaters
Source: Front Microbiol. 2020 Sep 24;11:555795. doi: 10.3389/fmicb.2020.555795 (PMC7541896; doi:10.3389/fmicb.2020.555795)
Supplement: TABLE S1 — The information of samples (sample ID, Sample Type, Date Location, Latitude and longitude and environmental Factors). [file Table_1.doc]

**Supplementary Table 1.** The information of samples (sample ID, Sample Type, Date Location, Latitude and longitude and environmental Factors).

| Latitude  and longitude | Environment | Glass | PFU | Water column | Water column | Date | Temperature (^o^C) | DO (mg/L) | pH | COD (mg/L) | TP (mg/L) | TN (mg/L) | AN (mg/L) | Salinity (‰) |
| --- | --- | --- | --- | --- | --- | --- | --- | --- | --- | --- | --- | --- | --- | --- |
| 23.131213N  113.348558 E | Lake | G1 | P1 | W1 |  | 2015.10.28 | 28.0 | 5.70 | 7.39 | 29.8 | 0.360 | 3.4 | 0.212 | < 0.2 |
|  |  | G2 | P2 | W2 |  | 2015.10.30 | 27.5 | 5.29 | 7.32 | 22.4 | 0.230 | 0.9 | 0.256 | < 0.2 |
|  |  | G3 | P3 | W3 |  | 2015.11.03 | 23.0 | 5.67 | 7.27 | 36.6 | 0.310 | 0.2 | 0.286 | < 0.2 |
|  |  | G4 | P4 | W4 |  | 2015.11.07 | 25.6 | 4.38 | 7.15 | 39.5 | 0.350 | 1.3 | 0.360 | < 0.2 |
|  |  | G5 | P5 | W5 |  | 2015.11.11 | 24.9 | 2.58 | 7.05 | 32.4 | 0.300 | 3.1 | 0.367 | < 0.2 |
|  |  | G6 | P6 | W6 |  | 2015.11.17 | 25.6 | 7.82 | 7.52 | 25.0 | 0.190 | 0.7 | 0.286 | < 0.2 |
|  |  | G7 | P7 | W7 |  | 2015.11.24 | 25.0 | 5.19 | 7.19 | 34.0 | 0.190 | 1.1 | 0.347 | < 0.2 |
| 23.112307N  113.330405 E | River | GR1 | PR1 | WH1 | WL1 | 2016.01.07 | 20.5 | 0.25 | 6.98 | 98.1 | 1.240 | 16 | 16.100 | < 0.2 |
|  |  | GR2 | PR2 | WH2 | WL2 | 2016.01.09 | 19.8 | 0.21 | 6.86 | 67.3 | 1.410 | 16.5 | 13.500 | < 0.2 |
|  |  | GR3 | PR3 | WH3 | WL3 | 2016.01.13 | 19.5 | 0.19 | 6.96 | 77.0 | 1.331 | 15 | 12.900 | < 0.2 |
|  |  | GR4 | PR4 | WH4 | WL4 | 2016.01.17 | 19.4 | 0.39 | 6.99 | 113.0 | 1.227 | 15.3 | 12.000 | < 0.2 |
|  |  | GR5 | PR5 | WH5 | WL5 | 2016.01.21 | 14.3 | 1.21 | 7.01 | 54.5 | 0.933 | 13.7 | 15.600 | < 0.2 |
